# Radiotherapy treatment in cancer control and its important role in Africa

**DOI:** 10.3332/ecancer.2019.942

**Published:** 2019-07-25

**Authors:** Ntokozo Ndlovu

**Affiliations:** University of Zimbabwe College of Health Sciences, Mazowe Street, PO Box 178, Avondale, Harare, Zimbabwe

**Keywords:** radiotherapy, cancer control, access, neoplasms, Africa

## Abstract

Radiotherapy, although an essential and cost-effective treatment for cancer, has a low uptake on the African continent. Common cancers treated in the region need radiotherapy as a component of therapy at some point in the course of the management of their disease. Within Africa, around half of the countries have no radiotherapy services and most of those that have cannot adequately cover the population which needs them.

To redress inequitable access, advocacy at all levels is needed for acceptance of radiotherapy and the important role it plays in cancer control. The relatively high cost of setting up radiotherapy services needs to be balanced with the long-term benefits of having a well-proven treatment modality that can be accessible to a large number of cancer patients at reasonable cost. Low healthcare funding in the region is a possible hindrance to access to radiotherapy.

Consideration of the appropriateness of technology to be procured when setting up such facilities is important. It should not, however, be simply a case of gamma versus photon teletherapy. The level of training of staff, quality evidence-based treatment provision and infrastructural considerations need to be taken into account. Where facilities are available, low radiotherapy utilisation rates are common and should be corrected. Good practice regarding quality assurance is important. Local research to test regional and adopted practices should be promoted to provide local evidence.

**Conclusions:**

With an imminent cancer epidemic, the scarcity of radiotherapy facilities in Africa is a cause for concern. The measures to correct this should include a multipronged approach that addresses specific needs for individual countries. Radiotherapy is an essential, cost-effective treatment for cancer and its availability must be given priority alongside the management interventions for other common diseases and conditions.

## Background

There are a number of commonly used, well-established treatment modalities for cancer therapy, such as radiotherapy and systemic therapies, including chemotherapy and surgery. Of these, radiotherapy is the least accepted, understood and utilised in Africa. In the background of this is a globally inequitable distribution of radiotherapy facilities whereby low- to middle-income countries are disadvantaged through a lack of or inadequate radiotherapy treatment services. In high-income countries, one radiotherapy machine is available for every 120,000 people and in middle-income countries, one machine serves over 1 million people [1]. This scenario dramatically changes in low-income countries where about 5 million or more people on average rely upon a single radiotherapy machine, as is the case with a large proportion of African countries, such as Nigeria, Ethiopia and the Democratic Republic of Congo amongst others. There is no access at all to radiotherapy treatment in 51 countries worldwide and about half of these are in Africa such as Malawi, Burundi, Lesotho and Chad [2].

The incidence of cancers in which radiotherapy is a major part of the management such as cervical and breast cancers that affect women is high in the region and so is that of head and neck cancers and prostate cancer where radiotherapy is much needed for treatment. In these settings, patients mostly present with late stage disease such that the role of surgery and common systemic therapies is minimised This largely transfers the burden of the resultant palliative treatment for the advanced cancers to the scarce radiotherapy treatment services [3, 4].

Within the African continent itself, there are many contrasts regarding availability and access to radiotherapy in the individual countries. A decline in the availability of this treatment modality in some countries, such as Madagascar and Cameroon, has been witnessed. Other countries, such as Egypt, Algeria and South Africa, provide more modern facilities which are rapidly expanding and improving their radiotherapy treatment portfolios [5, 6]. It is, therefore, apparent that a one-size-fits-all approach cannot address the needs for equitable access to radiotherapy services in Africa due to diversity in economic development, culture and differences in healthcare delivery systems across the vast continent of Africa. What remains apparent, however, is the need to develop and scale up radiotherapy accessibility in Africa, as it is glaringly inadequate and lagging behind the rest of the world.

## Improving access to quality radiotherapy treatment facilities in Africa

First and foremost, advocacy at all levels is needed for the recognition of the important and irreplaceable role that radiotherapy plays in cancer treatment. Radiotherapy is an essential component in the management of cancer patients for both cure and palliation, with up to 40% of cancers cured by radiotherapy alone compared to about 11% cured by chemotherapy alone [7]. It is well known that radiotherapy is the most cost-effective tool for cancer management compared to other modalities of treatment.

Radiotherapy facilities vary in size and technological level of equipment and it must be acknowledged that initial capital expenditure can be substantial, consisting of upfront and operational costs. The low health budgets in most African countries can, therefore, be a significant hindrance to establishing a good radiotherapy treatment infrastructure. It must, however, be noted that a radiotherapy unit, once established, is capable of treating large numbers of patients around the clock with reasonable running costs.

According to the WHO (World Health Organization) African Region Expenditure Atlas (2014), there was progress in mobilisation of government resources for health in general over a ten-year period of 2002 to 2012. In 2002, no African country had general government health expenditure (GGHE) as a share of gross domestic product that was more than 5%, but by 2007, four countries had achieved this target and this increased to seven by 2012. This low GGHE was attributed to the fact that many African countries have limited capacities to raise public revenues due to a number of reasons [8]. The low regional GGHE negatively affects the development of health infrastructure such as is needed for radiotherapy delivery.

Access to radiotherapy services has various dimensions, which include availability of the service, geographical accessibility, affordability, accommodation and awareness on the part of physicians and patients [2]. The radiotherapy utilisation (RTU) rate measures the proportion of cancer patients requiring at least one treatment course of radiotherapy during their disease process. In developed countries, the RTU rate is approximately 50%, meaning that 50% of patients diagnosed with cancer will require radiotherapy treatments at least once at some stage in the disease process. In developing countries, it is estimated that the optimal RTU rate should be higher and may reach 70%–80%. The higher rates are attributed to lack of prevention, screening programmes and limited oncological surgical services resulting in a high prevalence of advanced disease mostly needing radiotherapy as treatment of choice [1].

It has, however, been shown in reviews that the actual RTU rate in low-income countries is between 25% and 40%. This variation from the expectation has been attributed to constraints in diagnosing, reporting and referral for cancer treatment. It therefore follows that improvements in cancer diagnosis, registration and general healthcare infrastructure would go a long way towards improving the RTU rate in Africa [1].

The WHO also reported in 2012 that in Africa, only 10 countries out of 47 had an out-of-pocket (OOP) health expenditure as a percentage of total health expenditure (THE) that was less than 20%. It was also noted that 21 countries out of 47 had an OOP expenditure that was more than 40% of the THE, implying that treatment costs are high and radiotherapy may not be affordable to most cancer patients. Reducing financial barriers to accessing health services when needed is one of the top goals of universal health coverage. According to this report, it therefore calls upon governments to develop comprehensive/improve policies and strategies for health financing, promote prepayment mechanisms to cover the whole population and implement public equity funds to cover the health costs of people who are not able to contribute [8].

**Figure 1.**
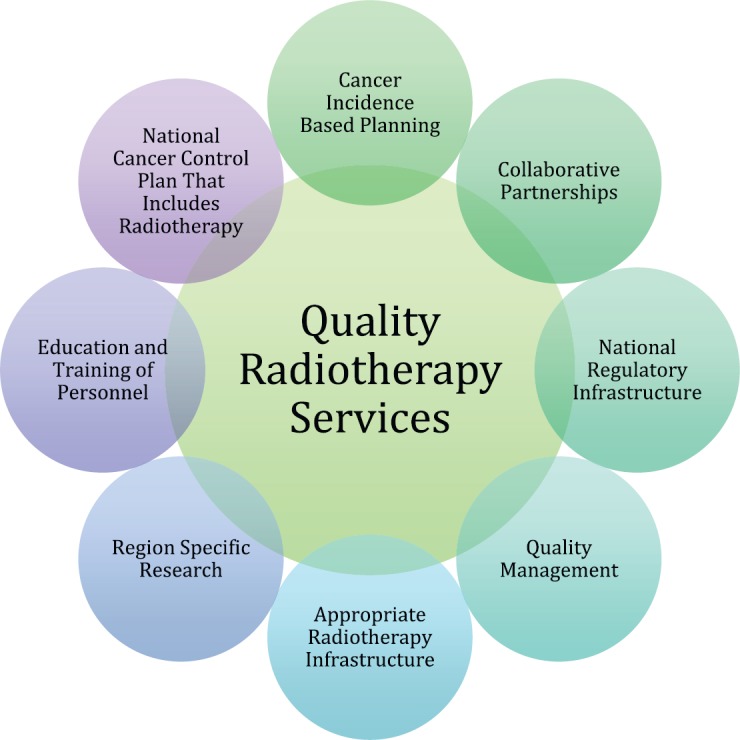
Components for structuring quality radiotherapy service provision.

In view of all of these considerations, it is apparent that the solutions to access to radiotherapy should be multipronged and addressed within the context of a national cancer control plan (see Figure 1). The political high-level United Nations declaration on the prevention and control of non-communicable diseases includes cancer and should be leveraged towards improving access to radiotherapy treatment in all United Nations member states [9].

There is a general lack of knowledge and fear of radiotherapy treatment by the public in Africa. This is largely driven by limited awareness and education on cancer, its causes and various treatment modalities. Some cultural practices may promote mysticism about cancer and its management that leads to reduced acceptance and uptake of radiotherapy as a standard form of cancer treatment. There is a common belief of linking cancer to spiritual issues such that the preferred default health seeking behaviours for cancer patients are those of looking to alternative rather than mainstream forms of treatment [10]. Also, since patients present with late-stage disease, which is a cause of poor survival even with the best of interventions, fear of radiotherapy may stem from the association of the intervention with demise that may usually follow shortly after palliative radiotherapy treatment.

The comprehensive approach to cancer control is still the most effective in ensuring that the role of radiotherapy is well articulated, integrated and planned in the most holistic manner. In principle, successful prevention of cancer would ease the burden of cancer overall and in turn the need of investing in large capacity radiotherapy facilities would be reduced. Early detection would ensure that cancers are diagnosed early and treated when there is the most benefit for curative therapy and thus improve further the justification of investing in radiotherapy treatment facilities [11]. The impression held by many in Africa of radiotherapy being the cause of bad outcomes in cancer would then diminish. This would possibly be followed by an increased acceptance of this treatment intervention within African communities.

The components of diagnosis and treatment for cancer share a lot in common as they require highly skilled personnel and technologically advanced equipment. Good national cancer control plans with adequate funding of these two can be the main key to service delivery in cancer management. The infrastructure needed has to be planned such that it is easily accessible to patients unlike what is currently most commonly the case where most such services are centralised, expensive and patients have to travel long distances to access them.

## Training of radiotherapy personnel in the African context

Indeed, for radiotherapy treatment, it is known that the optimal use of such services, in terms of effectiveness and safety, occurs when supporting accessories and trained staff complement it [1]. Manpower shortages of radiotherapy personnel are a global phenomenon that is more pronounced in Africa. It has been shown that training programmes for radiotherapy personnel in the region are few and inadequate. Established training programmes in sub-Saharan Africa can be found in countries, such as Tanzania, Zimbabwe, Ghana, South Africa Egypt, Morocco and Zambia with most countries relying on external training [12]. Sending trainees to other continents has not yielded the desired results of improved human resource self-sufficiency, as trained professionals are unlikely to return to their country of origin after training favouring to remain where they have been trained. This is likely due to lack of effective staff retention schemes and poorly equipped centres that do not match the knowledge to be applied by staff trained in highly equipped facilities.

Human resource shortages in radiotherapy affect Medical Physicists more than other radiotherapy personnel. This is further compounded by a lack of recognition of the medical physics profession in most African countries, which further promotes the flight of skills from where they are most needed. Regional and international collaboration is needed to ensure that radiotherapy personnel in Africa are well trained and retention schemes are in place to ensure that centres are adequately staffed to deliver this essential service [5, 6, 12].

Once trained, there is a need to ensure that staff keep up with new knowledge and trends in the field through continuing education. The inadequate staffing of most radiotherapy centres in the region negatively affects the promotion of a culture of continuing health education as more time is spent on clinical work and there is less peer interaction. Attending conferences and workshops can be expensive as most are held in far off locations. The African Radiation Oncology Network is one initiative that has demonstrated that it is possible to bridge the gap of peer to peer interactions by offering a platform for case discussion and various educative presentations through telemedicine [13].

## Setting up radiotherapy treatment facilities

In setting up radiotherapy treatment facilities, the choice of treatment machine technologies whilst important should not cloud the preparatory stages. The debate of cobalt teletherapy versus linear accelerators has to be considered in an informed evidence based manner [14]. Generally, equipment chosen should not limit the modern day practice of radiotherapy whilst taking into consideration issues of security of sources, electricity supply, transportation, recruitment, level of training and retention of staff [7]. There are many theoretical opinions on what radiotherapy equipment is ideal in the African context but there is little scientific analysis that takes into account the prevalent cancers, the population factors, the physics of radiotherapy, patient factors and circumstances under which the equipment is used.

Newer technologies and techniques have the well-recognised role of delivering uniform doses to the tumour whilst increasingly sparing normal tissues around the tumour. This translates into reduced side effects and improved outcomes. With increased awareness and uptake of screening, increasing numbers of patients in Africa will be treated with curative intent. It is, therefore, encouraging that uptake of newer technologies is also increasing in Africa as individual countries expand their services. With increasing complexity of treatment, however, more treatment time is needed and this can exert strain on scarce treatment resources utilised by large numbers of patients. Striking a balance between these without compromising treatment outcomes is an important factor. Consideration of these issues may need to be taken into account by many countries in Africa that are currently expanding radiotherapy portfolios.

Equipment maintenance can be a major drawback for radiotherapy service provision in Africa. It may seem that photon therapy gives longer downtime than gamma therapy but very long downtime has been experienced in Uganda and Madagascar with cobalt teletherapy equipment. Conversely in Nigeria, very long periods of downtime have been experienced with linear accelerators showing that downtime is not simply determined by the type of equipment available. Maintenance contracts are key in reducing downtime, be it ensuring timely change of gamma source or having engineering back up and maintaining a constant supply of electricity. The role of linear accelerators in the delivery of high-quality treatments in Africa should not be downplayed but all concerned parties should prioritise proper support and maintenance of this type of equipment [15, 16].

Brachytherapy services are equally, if not even more important to the African continent but are scarce. Cervical cancer is five times more common in Africa than in the United States of America and other developed countries. Locally advanced cervical cancer, the current most commonly occurring state of the disease in Africa, cannot be cured without intracavitary brachytherapy [7, 18]. It, therefore, becomes mandatory that cancer treatment facilities in sub-Saharan Africa be planned with brachytherapy facilities in mind. The choice of source should be well considered in terms of frequency of change to avoid periods of lack of brachytherapy facilities. Most African countries are now opting for the use of high-dose-rate brachytherapy using cobalt sources that have a long half-life, short treatment times and, therefore, are economically viable without compromising outcome [19]. This has considerably improved the availability and efficiency of delivering brachytherapy services.

Finally, robust radiotherapy services cannot be realised without full government support. First must be the realisation at government level of the cancer epidemic that is sweeping through Africa and the need to take action. This should be followed by adequate planning and funding of cancer-control programmes that are appropriate to the regional context from prevention to end-of-life issues and survivorship. Radiotherapy has an important and irreplaceable role in this and needs to be made available to all cancer patients in an equitable manner.

## Quality management in radiotherapy

In offering radiotherapy services, it is equally important to promote the uptake of quality management in radiotherapy. With understaffing and high patient numbers in many African centres, there is less time devoted to quality assurance, which is essential to ensure high standards and safety of radiotherapy treatment. Quality management, therefore, needs to be emphasised, prioritised and taught in all operational centres that may have been set up without such consideration in mind. As new centres are established, it has to be ensured that minimum quality assurance standards are in place prior to operation. For full implementation of this, it is imperative that effective national regulatory infrastructure is in existence and is geared towards ensuring radiation safety in all aspects of the use of ionising radiation, including radiotherapy practice [20]. The quality-assurance aspect of radiotherapy service delivery is gaining increasing uptake in Africa as newer centres and technologies are coming up and regulatory structure is strengthening.

## Cancer research in Africa

Strengthening of high-quality cancer research is a must for Africa, as most evidence-based interventions are not from evidence generated within the continent [21]. Research needs to be sufficiently diversified to address management of cancers common to the region, interactions of cancer and other diseases common to Africa such as HIV-AIDS as well as other important local concerns. An example is cancer of the cervix, which is very common in Africa, and is treated with lower energy teletherapy machines in women who are usually of larger physique, and known to have generally poorer outcomes. Interrogation of treatment techniques and dose distributions achievable in such settings can be done to determine if acceptable where currently, the recommendation is to use 10 MV energies to treat the pelvis. It may be important to obtain evidence whether choice of treatment energies might or might not be one of the many contributors to poorer outcomes in these patients.

## Conclusion

Radiotherapy is an essential cancer treatment whose low uptake in the African continent remains of concern. An increase in new centres and application of newer technologies is, however, noted to be on the rise. Multipronged innovative strategies are needed to urgently correct this situation of limited radiotherapy services availability that is occurring in the face of an imminent regional cancer epidemic. These strategies need to be tailored for each individual country according to local need.

Cancer treatment should not be perceived as taking second place to infection control or maternal and child health where healthcare provision is concerned as this is an important reason why radiotherapy remains a non-priority in Africa. The rather misconceived perception of radiotherapy as being a luxury that is expensive to set up should be dispelled.

Placed in the correct perspective, radiotherapy is an essential cost-effective treatment if it is planned and utilised effectively. It remains for governments and other healthcare planners in the region to actively include this form of treatment in national cancer control planning. Advocacy, education, awareness and reaching out across cultural boundaries to advance the cancer-control agenda ahead of the rising cancer epidemic is important and must include lobbying for acceptance of radiotherapy treatment in Africa.

## Conflicts of interest

The author declares that she has no conflict of interest.

## Funding

The writing of this manuscript has not been funded from any source.
